# Screening for latent infectious disease in patients with alopecia areata before initiating JAK inhibitors therapy: a single-center real-world retrospective study

**DOI:** 10.3389/fmed.2023.1287139

**Published:** 2023-10-18

**Authors:** Jundong Huang, Zixin Tan, Yan Tang, Wei Shi

**Affiliations:** Department of Dermatology, Hunan Key Laboratory of Aging Biology, Xiangya Hospital, Central South University, Changsha, China

**Keywords:** alopecia areata, Janus kinase inhibitors, screening, tuberculosis, hepatitis

## Abstract

**Introduction:**

Although there is growing evidence supporting the effectiveness of Janus kinase (JAK) inhibitors in treating alopecia areata, the high rate of recurrence following drug discontinuation has led to prolonged treatment courses and raised concerns about long-term safety. In clinical practice, caution should be exercised while using JAK inhibitors for various indications, and a comprehensive pre-treatment screening.

**Methods:**

This study presents an analysis of screening data collected from real-world settings before the initiation of Janus kinase inhibitors in patients with alopecia areata. Investigators collected retrospective medical data characterizing patients’ screening data. Data on demographic and clinical data, including age, sex, disease duration, severity of alopecia tool scale, history of prior treatment, and treatment regimen were recorded.

**Results:**

In this cohort (*N* = 218), JAK inhibitors were initiated for 163 of 218 (74.8%) alopecia areata patients. The numbers of patients positive for antinuclear antibodies, hepatitis B surface antigen, hepatitis C virus antibodies, human immunodeficiency virus antibody, treponema pallidum hemagglutination assay, and thyroid-stimulating hormone were 32 (32/176), 10(10/218), 0 (0/218), 0 (0/218), 3 (3/218) and 9 (9/176), respectively. The number of patients with T-cell spot positive or imaging of the chest indicating tuberculosis was 37 (37/218).

**Disccusion:**

Our data provide additional information on the safety profile of JAK inhibitors in patients with alopecia areata. As such, it is necessary and crucial to screen for JAK inhibitors before it is used, particularly for individuals with a high risk of tuberculosis, hepatitis B, and other infections.

## Introduction

Alopecia areata (AA) is a common autoimmune dermatosis characterized by nonscarring hairless patches that can involve most commonly the scalp or any hair-bearing part of the body (1), affecting approximately 0.1 to 0.2% of the population (2). With a recurrent or persistent course, AA can cause a severely reduced quality of life (3).

Although multifactorial in etiology, the mechanisms leading to AA are not fully understood but likely involve a combination of genetic predisposition and environmental triggers, which may disturb the immune balance of the follicle niche and destroy the immune privilege, eventually leading to the auto-immune attack of the follicle bulb cells mediated by T cells (4). Evidence from the studies on mouse models of AA has shown that the positive feedback loop of interferon (IFN)-gamma and interleukin (IL)-15 activate target immune cells and amplify the inflammatory response *via* the JAK–STAT signaling pathway (5).

The management of AA is notoriously challenging, with high rates of therapeutic failures or relapses. However, in the past decade, the JAK inhibitors have revolutionized the treatment of AA. Among them, baricitinib and ritlecitinib have been approved by the US FDA as standard therapies for treating severe alopecia areata cases (6–8). They have improved the overall therapeutic outcomes of AA treatment and allowed patients to pursue a hair-normal life, especially for patients who have previously failed other systemic treatments (9). Unfortunately, due to drug availability and other reasons, the choice of JAK inhibitors in China is quite less. Currently, the only approved treatment for AA in China is baricitinib. Moreover, aside from the impressive treatment effects, clinicians still expressed concerns about the potential activation of latent infectious issues resulting from JAK inhibitors use, including tuberculosis (TB), hepatitis B virus (HBV), human immunodeficiency virus (HIV), etc.

To standardize the clinical application of JAK inhibitors, a consensus statement, formulated by 29-person experts in multiple disciplines, was published in 2020 (10). It recommends pre-treatment screening including routine laboratory tests (full blood count and blood biochemistry), hepatitis virus testing for HBV and hepatitis C virus (HCV), HIV testing, tuberculosis infection testing before initiation of JAK inhibitors. However, no data on the screening under JAK inhibitors in real-world settings have been available to date, and the evidence is lacking on the necessity of screening in patients prior to administering this class of small molecule drugs. Here, we conducted a retrospective study to describe the screening data before the initiation of JAK inhibitors for AA and aim to provide supporting evidence for the standardization of the clinical use of JAK inhibitors.

## Methods

We conducted a retrospective study of all patients with AA prepared to treat with JAK inhibitors at our institution between February 2021 and October 2022. The study was approved by the institutional research ethics boards of Xiangya Hospital, Central South University (Changsha, China); approval number: 202303043.

The diagnosis of AA was confirmed by at least two dermatologists based on typical skin manifestations and results of dermoscopy. Patients with atypical manifestations (such as diffuse alopecia areata) were included only if confirmed by histologic findings. Patients with the unavailability of complete data were excluded. Patients who were pregnant or breast feeding or attempting to become pregnant were also excluded. All patients underwent screening including complete blood count, serum biochemical parameter, hepatitis B surface antigen (HBsAg), hepatitis C virus (HCV) antibodies, human immunodeficiency virus(HIV)antibody, treponema pallidum hemagglutination assay (TPHA), antinuclear antibodies (ANA), thyroid-stimulating hormone (TSH), T-cell spot (T-spot) test, and chest computed tomography (CT) or chest x-ray film at baseline. Treatment was initiated based on shared decision-making between the patient and the medical specialist and regular follow-up laboratory testing (including complete blood count, lipid profiles, and liver and renal functions) was conducted in patients who continued treatment.

For each patient, demographic and clinical data, including age, sex, disease duration, extra-scalp manifestation, severity of alopecia tool (SALT) scale (11), history of prior treatment, and treatment regimen were recorded.

### Statistical analysis

Descriptive statistics were summarized as number, percentage, mean and standard deviation (mean ± SD), and median and range.

## Results

Of 232 patients evaluated, 218 patients were included in the study. Their characteristics are shown in Table 1. The sex distribution of study patients was 81 men (37.2%) versus 137 women (62.8%), median age was 29.0 years (IQR 20.0–40.5). The disease duration ranged from 2 to 277 months, with 59 patients more than 60 months. Except for 2 patients with<25% hair loss, most patients met the diagnostic criteria of moderate to severe AA, among which 61 patients were dignosed as alopecia totalis or alopecia universalis. In all, patients with body hair loss and nail involvement represented 109(50.0%) and 29(13.3%), respectively. In addition, systemic steroids and immunosuppressive agents were formerly used by 110(50.5%) and 10(4.6%), respectively.

**Table 1:** Patient characteristics.

Variable	*n* = 218
Age, year, median (range)	29 (6, 66)
*Sex, n (%)*	
female	81 (37.2)
male	137 (62.8)
Duration of disease, y, median (range)	25.5 (2, 277)
*Duration of disease, m, n (%)*	
<12	69 (31.7)
12 ~ 60	90 (41.2)
>60	59 (27.1)
*SALT subclass, n (%)*	
S1 = <25% hail loss	2 (0.9)
S2 = 25–49% hail loss	45 (20.6)
S3 = 50–74% hail loss	85 (39.0)
S4 = 75–99% hail loss	25 (11.5)
S5 = 100% hail loss	61 (28.0)
Body hair loss, n (%)	109 (50.0)
Nail involvement, n (%)	29 (13.3)
*Previous treatments, n (%)*	
Systemic steroids	110 (50.5)
Immunosuppressive agent^a^	10 (4.6)

a Including cyclosporine and methotrexate.

**Figure 1:**
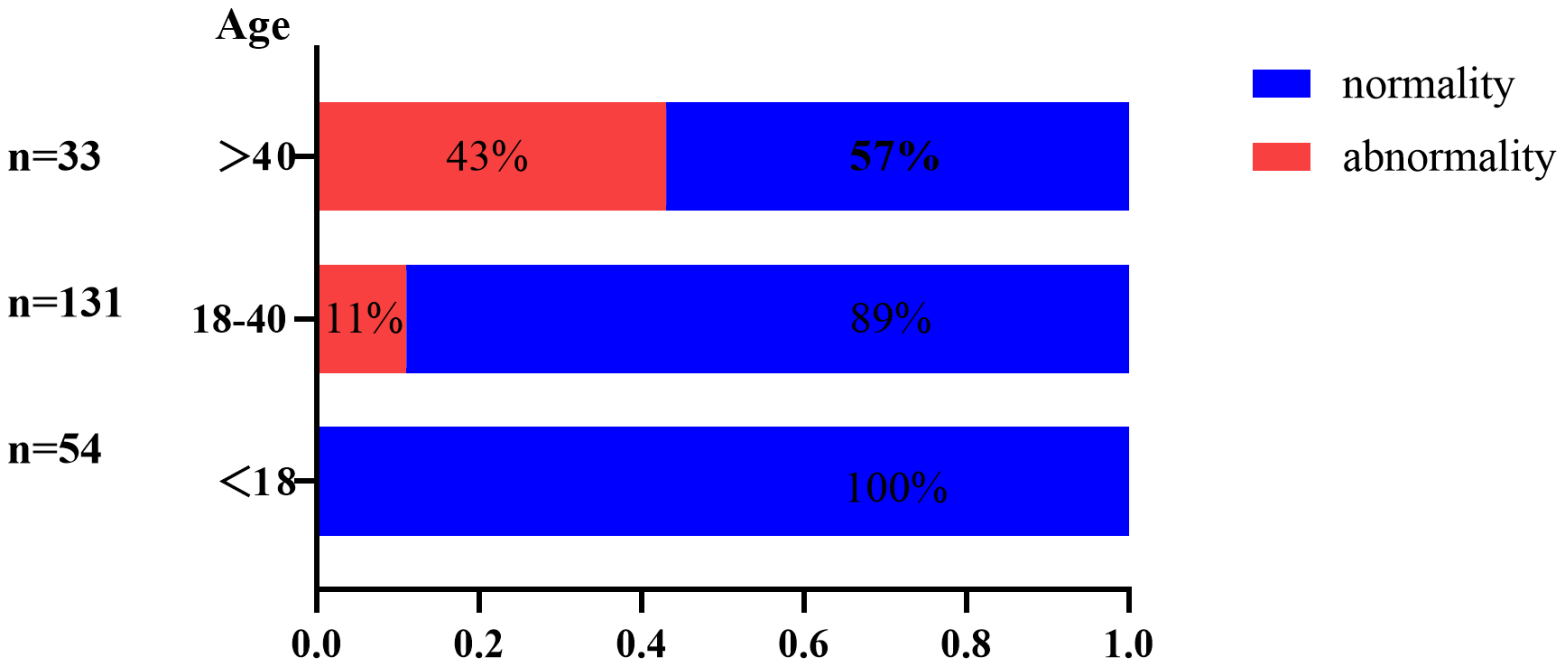
Screening abnormality rates for tuberculosis (%) in different age groups.

Abnormal screening results were shown in Table 2. All patients were screened for infection-related examinations. A total of 31 (14.2%) patients presented positive for T-spot, among which only 1 patient showed signs indicating tuberculosis on chest radiograph. Of those presented negative for T-spot (*n* = 187), 6 patients showed signs of tuberculosis infection on chest radiograph. However, no patients reported tuberculosis infection-related clinical manifestations. Figure 1 shows the age distribution of patients screened for tuberculosis infection. The infection rate was 0, 11 and 43% in the groups with <18, 18–40 and >40 age groups, respectively. Moreover, the number of patients positive for HBsAg, HCV, HIV, and TPHA were 10 (4.6%), zero (0%), zero (0%), and 3 (1.4%), respectively. Five of the ten patients who were HBsAg positive had HBV DNA quantification positive. With regard to the TPHA-positive patient, syphilis serum antibody titers were not detected.

**Table 2:** Abnormal screening results and treatment options of 218 patients.

Variable	Number	*n* (%)
*Infection-related blood tests*		
T-spot test	218	31 (14.2)
HBsAg	218	10 (4.6)
HCV antibodies	218	0 (0)
HIV antibodies	218	0 (0)
TPHA	218	3 (1.4)
*Immunity-related blood tests*		
TSH	176	9 (5.1)
ANA	176	32 (18.2)
ds-DNA antibody	176	1 (0.6)
*Imageological examination*		
CT^a^	218	17 (7.8)
*Treatment options*		
Tofacitinib	218	148 (67.9)
Baricitinib	218	10 (4.6)
Abrocitinib	218	3 (1.4)
Jaktinib	218	2 (0.9)
conservative management^b^	218	25 (11.5)
Oral corticosteroids	218	4 (1.8)
Immunosuppressive agents	218	12 (5.5)
Withdrawl	218	14 (6.4)

a Only meaningful reported results that affected treatment decisions were included.

b Including topical corticosteroids, intralesional steroids, and topical minoxidil.

HBsAg, hepatitis B surface antigen; HCV, hepatitis C virus; HIV, human immunodeficiency virus; TPHA, treponema pallidum hemagglutination assay; ANA, antinuclear antibodies; TSH, thyroid-stimulating hormone; CT, chest computed tomography.

A total of 176 patients were screened for immunity-related examinations. ANA was positive in 32 patients (18.2%), of which 26, 5 and 1 patients had antibody titers of 1:80, 1:160 and 1:320, respectively. Among the patients with antibody titers of 1:80, the numbers of speckled, nucleolar and speckled-nucleolar patterns were 20, 4 and 2, respectively. Anti-double-stranded DNA (ds-DNA) antibody positivity was detected only in one patient with an antinuclear antibody titer of 1:320, who was subsequently diagnosed with systemic lupus erythematosus. Thyroid-stimulating hormone (TSH) abnormalities were detected in 9 patients (5.1%), of which 8 patients had a significant increase in TSH.

Chest imageological examinations were screened in all patients. The number of positive results was 17 (7.8%). Of these, the results were pulmonary inflammation, tuberculosis infection, pulmonary 4A nodules, pulmonary 4B nodules and pulmonary hypertension in 5, 7, 1, 3 and 1 patient, respectively.

Treatment options were also shown in Table 2. JAK inhibitors, conservative management, oral corticosteroids, immunosuppressive agents and withdrawl were initiated for 163 (74.8%), 25 (11.5%), 4 (1.8%), 12 (5.5%) and 14 (6.4%), respectively. Among those received JAK inhibitor, the most commonly prescribed targeted therapies were Tofacitinib (*n* = 148), followed by Baricitinib (*n* = 10), Abrocitinib (*n* = 3), and Jaktinib (*n* = 2). Despite the rapid development of novel JAK inhibitors, tofacitinib remains an effective and the longest-used JAK inhibitor for AA. As such, we often tend to prescribe tofacitinib. The detailed information on the approval status of the above drugs is provided in Supplementary Table S1. What’s more, 36 patients abandoned JAK inhibitor due to signs indicating tuberculosis. Another reason patients discontinued JAK inhibitors is long-term security considerations.

## Discussion

Driven by the lack of study describing the screening data of JAK inhibitors, we evaluated the blood examination and chest imaging screening data before initiation of JAK inhibitors for AA, providing supporting evidence for standardizing the clinical application of JAK inhibitors in practice. Overall, JAK inhibitors were initiated for 163 of 218 (74.8%) AA patients. The most common reasons for patients failing screening were tuberculosis and hepatitis B. Notably, we followed a possible association between AA and lung cancer. The chest CT screened 4 patients with lung-RADS category 4 nodules, 1 of whom was ultimately diagnosed with lung adenocarcinoma.

Latent tuberculosis infection (LTBI) is a state in which the host presents with immunoreactivity to tuberculosis antigens but without clinical and radiologic evidence of tuberculosis disease (12). It is estimated that the lifetime risk of reactivation in people with LTBI is 5–10%, while some drugs can aggravate this process (13). Notably, JAK inhibitors may reactivate latent tuberculosis by inhibiting macrophage function and causing granuloma structure dissolution. Chen et al. investigated the rate of reactivation of tuberculosis development in rheumatoid arthritis patients undergoing oral tofacitinib therapy (14). Among 28,099 patients undergoing the therapy, 79 (0.28%) patients developed active tuberculosis. Fifty three of the 79 patients were T-spot negative before treatment, while 5 were T-spot positive and developed tuberculosis in the case of combined isoniazid treatment. The 2015 American guideline for the treatment of rheumatoid arthritis suggests that patients with T-spot positive can start immunosuppressants after completion of at least 1 month treatment for anti-tuberculosis (15). China ranks as having the third-highest tuberculosis burden of any country in the world which can reasonably explain our findings (16). In the present study, 13.76% (30/218) of patients with AA were detected as LTBI. This rate was slightly lower than that in a previous LTBI report in the general population in the rural regions of China (LTBI rate is 18.8% (3,955/21022)) (17). However, we should emphasize that the 13.76% does not represent the prevalence of LTBI in AA becasue the majority of patients receiving other treatment were not screened for TB in real-life experience. Amony the 30 patients of LTBI, only 1 patient who refused LTBI prophylaxis treatment received tofacitinib treatment and reexamination of chest CT showed no obvious changes after 6 months. Despite the disease tends for AA to be prevalent in younger patients, the data that observed in larger, long-term studies of patients with rheumatoid arthritis strongly suggest a link between activation of tuberculosis and JAKi (18, 19). In additional, our data showed that the proportion of tuberculosis infections detected among patients over 40 years is 43%, which decreased to 11% in the 18–40 age group. As such, we recommend screening for TB infections on a routine basis before treatment with JAK inhibitors, especially for people over 40 years. These patients should also be closely monitored during the JAK inhibitors therapy to identify new tuberculosis infection or reactivation of latent tuberculosis (19, 20).

Hepatitis B virus (HBV) infection is a major global health issue, with 257 million chronically infected individuals and 887 000 HBV-related deaths in 2015 (21). In patients have previously had HBV infection, hepatitis can develop due to reactivation of the virus by the use of immunosuppressants (22). Thus, it is recommended that hepatitis B virus surface antigen (HBsAg) or hepatitis B virus core antibody (HBcAb) should be performed before treatment. Wang et al. had investigated the long-term outcome of reactivation of HBV (rHBV) development in rheumatoid arthritis patients undergoing tofacitinib therapy. Two of the 64 (0.03%) HBcAb positive patients developed rHBV, while 2 of the 6 (33%) HBsAg positive developed rHBV. It is worth noting that combined antiviral therapy could signifficantly reduce viral reactivation. In this study, only 1 patient with HBsAg positive/HBV DNA negative were treated with oral tofacitinib combined with entecavir and no HBV reactivation was observed during the follow-up.

Previous studies have shown a link between AA and autoimmune diseases, in particular thyroid diseases, suggesting the same genetic background and pathogenesis of these diseases (23). However, the latest meta-analyses agree that (24, 25) AA is not correlated with thyroid dysfunction and routine screening for thyroid diseases is not recommended. A retrospective study on children with AA screened for thyroid function also support this view (26). Among all included subjects, 9 patients (5.1%) presented an abnormal TSH serum level, of which 8 patients showed an elevated TSH concentration. This is consistent with a previous study by Thomas et al. (27). Antinuclear antibodies (ANA) are a group of autoantibodies targeting various antigen components within cells, which can be characterized in a variety of autoimmune diseases (28). However, there is no consensus on the relationship between ANA and AA. In this study, the number of ANA positive patients was 32, among whom only 1 patient was subsequently diagnosed as systemic lupus erythematosus. Considering the experience on clinical practice and our cohort data, it is reasonable to screen for thyroid function abnormalities and other autoimmune disease in patients with AA. What’s more, it is not clear whether ANA or thyroid antibodies have an effect on the progresis of AA.

Currently, only a few studies have evaluated the association between AA and malignancy. A cohort study from Taiwan showed that the total cancer incidence of AA patients was slightly lower than that of the general population, especially in male patients (standardized incidence rate was 0.89). But the incidence of some types of malignancies, such as lymphoma (standardized incidence rate was 1.55), breast cancer (standardized incidence rate was 2.93), and urinary malignancies (standardized incidence rate was 2.95), was significantly higher, suggesting that there may be organ tendency between AA and cancer (29). However, a cohort study from Korea showed that the cancer incidence of AA patients is slightly higher than that of the general population (HR1.043; 95% CI1.022–1.065). After standardized age, sex and comorbidity, there are still significant differences in the risk of thyroid cancer, bladder cancer and prostate cancer (30). Further supporting evidence comes from another study showed the risk of cancer-related death of AA was higher than that of the general population. Especially, patients with alopecia universalis had a significantly higher risk of lung carcinoma-related death (HR2.16; 95% CI, 1.41–3.33) (31). In this study, a 32-year-old female with a lung-RADS category 4B nodule was diagnosed with lung adenocarcinoma after a biopsy. Our fingdings, along with previous research, suggest a possible association between AA and malignancy. Notably, JAK inhibitors may theoretically increase the risk of tumor occurrence or progression. However, there is currently no agreement on the cancer screening strategy before JAK inhibitors.

This study has some limitations, including a small sample size, a single-center study, and a single disease population. However, our study is the first to describe the screening data under JAK inhibitors, which has important implications for the awareness of the safety of JAK inhibitors. Additionally, other laboratory tests such as complete blood count, routine urinalysis, fasting blood glucose and blood biochemistry were not described. Moreover, we did not collect comprehensive data about other risk factors associated with cardiovascular risk (e.g., obesity, current smoking history, and family history of coronary heart disease of premature onset) and family history of malignancy. However, the study population is largely composed of young or middle-aged individual who are less affected by biases related to comorbidities. Future larger studies are needed to evaluate the cost–benefit of such a screening approach.

## Conclusion

In summary, we evaluated the screening data before initiation of JAK inhibitors for 218 AA patients in the real-world setting. The numbers of patients positive for ANA, HBsAg, HCV antibody, HIV antibody, TPPA and TSH were 32 (32/176), 10(10/218), 0 (0/218), 0 (0/218), 3 (3/218) and 9 (9/176), respectively. The number of patients with T-spot positive or pulmonary CT indicating tuberculosis was 37 (37/218). In developing countries, it is crucial to screen for JAK inhibitors before use, particularly for individuals with a high risk of tuberculosis, hepatitis B, and other infections.

## Data availability statement

The original contributions presented in the study are included in the article/Supplementary material, further inquiries can be directed to the corresponding author.

## Ethics statement

The studies involving humans were approved by the institutional research ethics boards of Xiangya Hospital, Central South University (Changsha, China). The studies were conducted in accordance with the local legislation and institutional requirements. Written informed consent for participation was not required from the participants or the participants' legal guardians/next of kin in accordance with the national legislation and institutional requirements.

## Author contributions

JH: Data curation, Writing – original draft. ZT: Data curation, Writing – original draft. YT: Data curation, Writing – original draft. WS: Writing – review & editing.

## References

[1] Gilhar A Etzioni A Paus R. Alopecia areata. N Engl J Med. (2012) 366:1515–25. doi: 10.1056/NEJMra1103442 22512484

[2] Strazzulla LC Wang EHC Avila L Lo Sicco K Brinster N Christiano AM. Alopecia areata: disease characteristics, clinical evaluation, and new perspectives on pathogenesis. J Am Acad Dermatol. (2018) 78:1–12. doi: 10.1016/j.jaad.2017.04.1141 29241771

[3] Liu LY King BA Craiglow BG. Health-related quality of life (HRQoL) among patients with alopecia areata (AA): a systematic review. J Am Acad Dermatol. (2016) 75:806–12. doi: 10.1016/j.jaad.2016.04.035 27436156

[4] Bertolini M McElwee K Gilhar A Bulfone-Paus S Paus R. Hair follicle immune privilege and its collapse in alopecia areata. Exp Dermatol. (2020) 29:703–25. doi: 10.1111/exd.14155 32682334

[5] Wang EHC Khosravi-Maharlooei M Jalili RB Yu R Ghahary A Shapiro J. Transfer of alopecia Areata to C3H/HeJ mice using cultured lymph node-derived cells. J Invest Dermatol. (2015) 135:2530–2. doi: 10.1038/jid.2015.176 25946709

[6] FDA Approves First Systemic Treatment for Alopecia Areata, (2022). Available at: https://www.fda.gov/news-events/press-announcements/fda-approves-first-systemic-treatment-alopecia-areata

[7] King B Zhang X Harcha WG Szepietowski JC Shapiro J Lynde C. Efficacy and safety of ritlecitinib in adults and adolescents with alopecia areata: a randomised, double-blind, multicentre, phase 2b-3 trial. Lancet. (2023) 401:1518–29. doi: 10.1016/S0140-6736(23)00222-2 37062298

[8] King BA Craiglow BG. Janus kinase inhibitors for alopecia areata. J Am Acad Dermatol. (2023) 89:S29–32. doi: 10.1016/j.jaad.2023.05.049 37591562

[9] Liu LY Craiglow BG Dai F King BA. Tofacitinib for the treatment of severe alopecia areata and variants: a study of 90 patients. J Am Acad Dermatol. (2017) 76:22–8. doi: 10.1016/j.jaad.2016.09.007 27816293

[10] Nash P Kerschbaumer A Dörner T Dougados M Fleischmann RM Geissler K. Points to consider for the treatment of immune-mediated inflammatory diseases with Janus kinase inhibitors: a consensus statement. Ann Rheum Dis. (2021) 80:71–87. doi: 10.1136/annrheumdis-2020-218398 33158881 PMC7788060

[11] Olsen EA Hordinsky MK Price VH Roberts JL Shapiro J Canfield D. National Alopecia Areata Foundation. Alopecia areata investigational assessment guidelines--part II. National Alopecia Areata Foundation. J Am Acad Dermatol. (2004) 51:440–7. doi: 10.1016/j.jaad.2003.09.032 15337988

[12] Zhou G Luo Q Luo S Teng Z Ji Z Yang J. Interferon-γ release assays or tuberculin skin test for detection and management of latent tuberculosis infection: a systematic review and meta-analysis. Lancet Infect Dis. (2020) 20:1457–69. doi: 10.1016/S1473-3099(20)30276-0 32673595

[13] Cantini F Nannini C Niccoli L Petrone L Ippolito G Goletti D. Risk of tuberculosis reactivation in patients with rheumatoid arthritis, ankylosing spondylitis, and psoriatic arthritis receiving non-anti-TNF-targeted biologics. Mediat Inflamm. (2017) 2017:8909834. doi: 10.1155/2017/8909834 PMC5474286 28659665

[14] Cantini F Blandizzi C Niccoli L Petrone L Goletti D. Systematic review on tuberculosis risk in patients with rheumatoid arthritis receiving inhibitors of Janus kinases. Expert Opin Drug Saf. (2020) 19:861–72. doi: 10.1080/14740338.2020.1774550 32552289

[15] Singh JA Saag KG Bridges SL Jr Akl EA Bannuru RR Sullivan MC. 2015 American College of Rheumatology Guideline for the treatment of rheumatoid arthritis. Arthritis Rheumatol. (2016) 68:1–26. doi: 10.1002/acr.22783 26545940

[16] Chakaya J Petersen E Nantanda R Mungai BN Migliori GB Amanullah F. The WHO global tuberculosis 2021 report - not so good news and turning the tide back to end TB. Int J Infect Dis. (2022) 124:S26–9. doi: 10.1016/j.ijid.2022.03.011 35321845 PMC8934249

[17] Gao L Bai L Liu J Lu W Wang X Li X. LATENTTB-NSTM study team. Annual risk of tuberculosis infection in rural China: a population-based prospective study. Eur Respir J. (2016) 48:168–78. doi: 10.1183/13993003.00235-2016 27230438

[18] Zhang Z Deng W Wu Q Sun L. Tuberculosis, hepatitis B and herpes zoster in tofacitinib-treated patients with rheumatoid arthritis. Immunotherapy. (2019) 11:321–33. doi: 10.2217/imt-2018-0113 30630365

[19] Ji X Hu L Wang Y Man S Liu X Song C. Risk of tuberculosis in patients with rheumatoid arthritis treated with biological and targeted drugs: meta-analysis of randomized clinical trials. Chin Med J. (2022) 135:409–15. doi: 10.1097/CM9.0000000000001948 35194004 PMC8869575

[20] Samuel C Cornman H Kambala A Kwatra SG. A review on the safety of using JAK inhibitors in dermatology: clinical and laboratory monitoring. Dermatol Ther (Heidelb). (2023) 13:729–49. doi: 10.1007/s13555-023-00892-5 36790724 PMC9930707

[21] WHO Hepatitis B key facts. (2021).Available at: https://www.who.int/news-room/fact-sheets/detail/hepatitis-b

[22] European Association for the Study of the Liver. EASL 2017 clinical practice guidelines on the management of hepatitis B virus infection. J Hepatol. (2017) 67:370–98. doi: 10.1016/j.jhep.2017.03.021 28427875

[23] Chen CH Wang KH Lin HC Chung SD. Follow-up study on the relationship between alopecia areata and risk of autoimmune diseases. J Dermatol. (2016) 43:228–9. doi: 10.1111/1346-8138.13165 26499292

[24] Kinoshita-Ise M Martinez-Cabriales SA Alhusayen R. Chronological association between alopecia areata and autoimmune thyroid diseases: a systematic review and meta-analysis. J Dermatol. (2019) 46:702–9. doi: 10.1111/1346-8138.14940 31197884

[25] Lee S Lee H Lee CH Lee WS. Comorbidities in alopecia areata: a systematic review and meta-analysis. J Am Acad Dermatol. (2019) 80:466–77. doi: 10.1016/j.jaad.2018.07.013 30031145

[26] Patel D Li P Bauer AJ Castelo-Soccio L. Screening guidelines for thyroid function in children with alopecia Areata. JAMA Dermatol. (2017) 153:1307–10. doi: 10.1001/jamadermatol.2017.3694 28973128 PMC5817442

[27] Thomas EA Kadyan RS. Alopecia areata and autoimmunity: a clinical study. Indian J Dermatol. (2008) 53:70–4. doi: 10.4103/0019-5154.41650 19881991 PMC2763714

[28] Agmon-Levin N Damoiseaux J Kallenberg C Sack U Witte T Herold M. International recommendations for the assessment of autoantibodies to cellular antigens referred to as anti-nuclear antibodies. Ann Rheum Dis. (2014) 73:17–23. doi: 10.1136/annrheumdis-2013-203863 24126457

[29] Chen CC Chang YT Liu HN Chen YJ. Cancer risk in patients with alopecia areata: a nationwide population-based matched cohort study. Cancer Med. (2018) 7:2153–9. doi: 10.1002/cam4.1448 29577672 PMC5943418

[30] Lee JH Song Y Do Han K Park YM Lee JY Park YG. Cancer risk by the subtype of alopecia. Sci Rep. (2018) 8:9748. doi: 10.1038/s41598-018-28142-1 29950587 PMC6021412

[31] Lee S Lee YB Kim BJ Bae S Lee WS. All-cause and cause-specific mortality risks associated with alopecia Areata: a Korean Nationwide population-based study. JAMA Dermatol. (2019) 155:922–8. doi: 10.1001/jamadermatol.2019.0629 31141109 PMC6547083

