# Supplementary material for: Screening for latent infectious disease in patients with alopecia areata before initiating JAK inhibitors therapy: a single-center real-world retrospective study
Source: Front Med (Lausanne). 2023 Oct 18;10:1287139. doi: 10.3389/fmed.2023.1287139 (PMC10619649; doi:10.3389/fmed.2023.1287139)
Supplement: Supplementary file 1 [file Table_1.pdf]

Supplement Table 1 The approval status and drug indications

	Baricitinib	Tofacitinib	Abrocitinib	Jaktinib***
US FDA-approved*	✔	×	×	×
China-FDA-approve*	✔	×	×	×
Registered indications**	Rheumatoid arthritis; The coronavirus disease-2019 (COVID-19); Alopecia areata	Rheumatoid arthritis; Ankylosing spondylitis; Psoriatic arthritis; Ulcerative colitis; Juvenile idiopathic arthritis	Atopic dermatitis	None

*: Approved indications for the treatment of alopecia areata

**: Based on US FDA-approved indications

***: Jaktinib is a new, small, orally administered multi-target JAK inhibitor that was independently developed in China and is currently in phase III clinical trials (NCT05255237; NCT05051761).
